# Choke Attack: Atypical Cardiomyopathy Subsequent to Postnasal Drip

**DOI:** 10.7759/cureus.2182

**Published:** 2018-02-11

**Authors:** Kantha R Kolla, Jaya M Mehta, Hari P Chaliki

**Affiliations:** 1 Cardiology, Mayo Clinic, Scottsdale, AZ; 2 Mayo Clinic, Scottsdale, Az, Mayo Clinic, Scottsdale, AZ

**Keywords:** angina, cardiomyopathies, heart contractility, hyperkinesis, takotsubo cardiomyopathy

## Abstract

Midventricular ballooning syndrome, an atypical presentation of takotsubo cardiomyopathy (TCM), presents with transient wall motion abnormalities of the midsegment of the left ventricle with apical sparing. In midventricular TCM, apical contractility is unaffected or may be hyperkinetic in contrast to the typical form of TCM. We report a case of atypical TCM, wherein the patient presented with chest pain following choking and coughing spells due to a postnasal drip.

## Introduction

Physical or emotional stress acts as a trigger for takotsubo cardiomyopathy [1]. The most common presentation in takotsubo cardiomyopathy is hypokinesis of apical left ventricular wall motion [2]. In our case, the patient developed a midventricular variant of takotsubo cardiomyopathy due to benign episodes of coughing and choking.

## Case presentation

**Figure 1:**
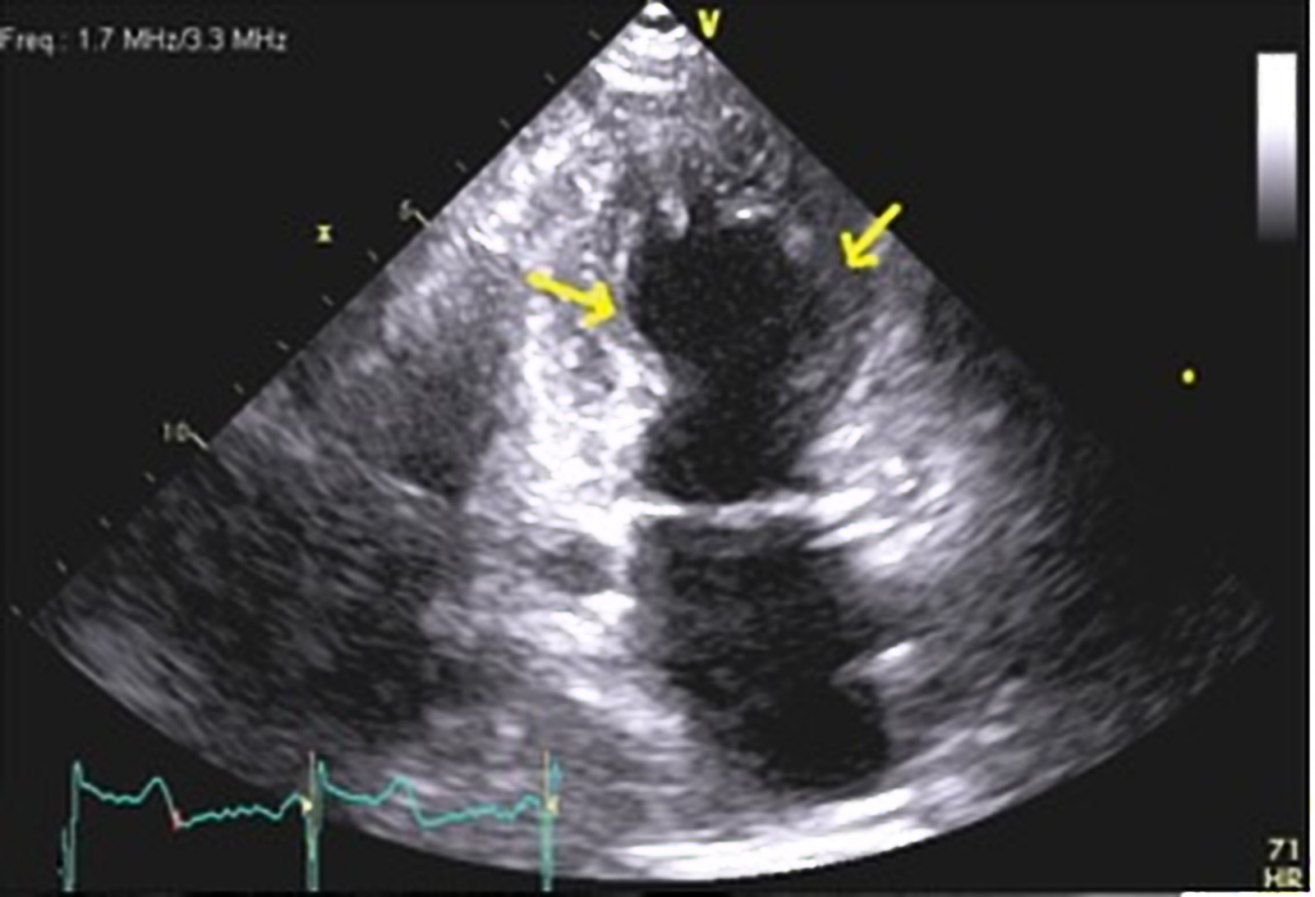
Transthoracic echocardiogram during systole, showing ballooning of the mid-left ventricular cavity

**Figure 2:**
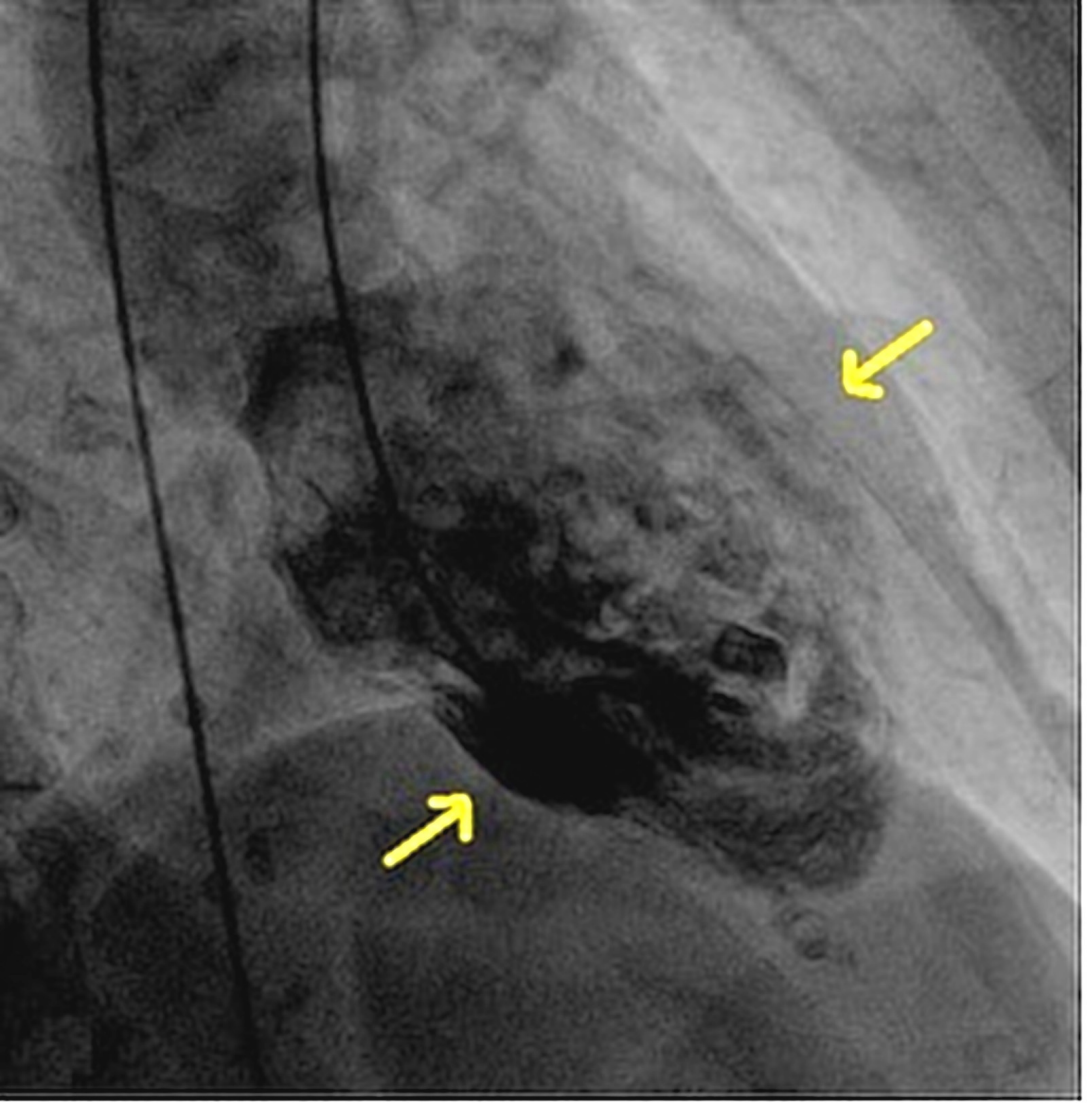
Left heart catheterization, showing ballooning of the mid-left ventricular cavity during diastole

**Figure 3:**
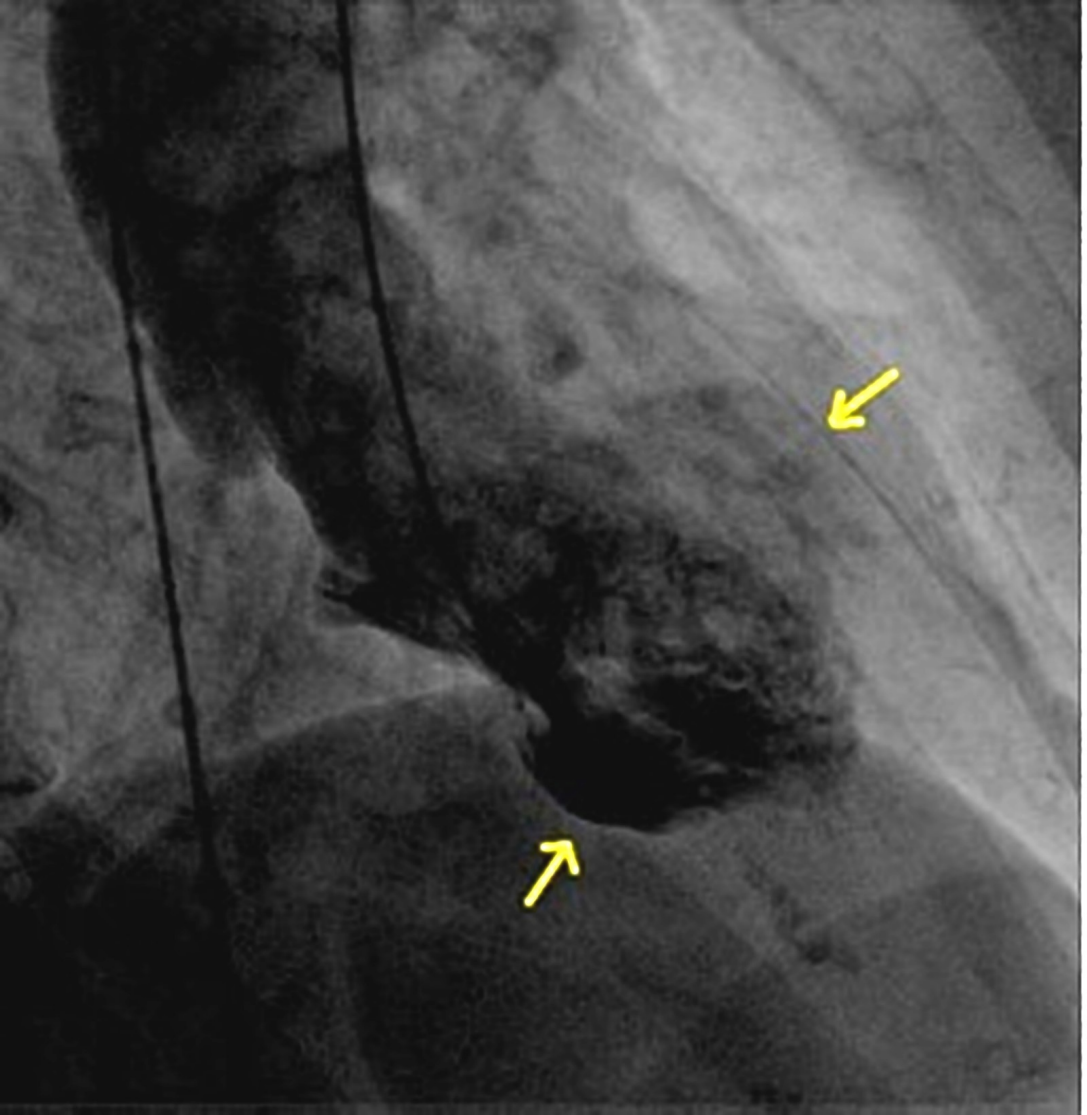
Left heart catheterization, showing ballooning of the mid-left ventricular cavity during systole

An 86-year-old woman with a past medical history of hyperlipidemia, osteoporosis, hypothyroidism, and papilloma tongue presented to the emergency department with chest pain and shortness of breath. This was preceded by an episode of coughing and choking spells due to a postnasal drip. She had episodes of anxiety and low mood for one year following the death of her life partner. On examination, the patient was alert and well-oriented. Her heart rate was 107 and blood pressure was 138/95. Electrocardiography showed atrial fibrillation and low voltage QRS. Cardiac biomarker troponin T levels were elevated to 0.1 mcg/L (reference range 0-0.03 mcg/l). A transthoracic echocardiogram showed findings of a reduction in the global left ventricular systolic function, an ejection fraction of 47%, and prominent mid anterolateral and inferolateral regional wall motion abnormalities (Figure 1, Video 1). Subsequently, left heart catheterization was done, which excluded significant coronary artery disease but showed a mid-left ventricular wall ballooning during diastole (Figure 2) and systole (Figure 3). Thus, echocardiography, left heart catheterization, and left ventricular angiogram findings (Video 2) suggest a midventricular variant of takotsubo cardiomyopathy (TCM), which is an atypical presentation. At the follow-up visit, the ECG showed a normal sinus rhythm and stress echocardiography showed a normal ejection fraction without regional wall motion abnormalities and improved left ventricular systolic function.

**Video 1:** Transthoracic echocardiography showing mid-left ventricular wall motion abnormalities

**Video 2:** Left ventricular angiography, demonstrating an abnormality in mid-ventricular wall motion

## Discussion

Takotsubo cardiomyopathy (TCM) is typically characterized by hypokinesis of apical ventricular wall motion. It was initially recognized in Japan in 1990. Midventricular ballooning syndrome, an atypical variant of TCM is characterized by hypokinesis of the midventricular segment [1]. The proposed pathogenesis of TCM includes: (1) catecholamine cardiac toxicity; (2) myocardial sympathetic innervation; (3) myocardial microvascular dysfunction; and (4) aborted myocardial infarction [3]. Based on the pattern of left ventricular wall involvement, there are four types of takotsubo cardiomyopathy: (1) inverted/reverse, (2) midventricular, (3) localized and (4) global type. The difference in the distribution, density, and sensibility of adrenergic receptors over the myocardium determines the area of hypokinesis. The area with the highest density of adrenergic receptors will be the most affected [4-5]. Emotional stress has been found to be one of the major triggers of TCM [6]. Our case highlights the fact that even benign conditions, such as choking and coughing spells, can trigger an emotional response that can lead to midventricular TCM.

## Conclusions

Prognosis is favorable for most patients with TCM. Our case illustrates that sudden stress, even due to generally benign episodes, such as choking and coughing, can lead to TCM.

## References

[1] Velankar P, Buergler J (2012). A mid-ventricular variant of takotsubo cardiomyopathy. Methodist Debakey Cardiovasc J.

[2] Zeb M, Sambu N, Scott P, Curzen N (2011). Takotsubo cardiomyopathy: a diagnostic challenge. Postgrad Med J.

[3] Chen W, Dilsizian V (2017). Exploring the pathophysiology of takotsubo cardiomyopathy. Curr Cardiol Rep.

[4] Pierard S, Vinetti M, Hantson P (2014). Inverted (reverse) takotsubo cardiomyopathy following cerebellar hemorrhage. Case Rep Cardiol.

[5] Kaoukis A, Panagopoulou V, Mojibian HR, Jacoby D (2012). Reverse takotsubo cardiomyopathy associated with the consumption of an energy drink. Circulation.

[6] Wittstein IS, Thiemann DR, Lima JA (2005). Neurohumoral features of myocardial stunning due to sudden emotional stress. N Engl J Med.

